# Blood Brain Barrier and Neuroinflammation Are Critical Targets of IGF-1-Mediated Neuroprotection in Stroke for Middle-Aged Female Rats

**DOI:** 10.1371/journal.pone.0091427

**Published:** 2014-03-11

**Authors:** Shameena Bake, Amutha Selvamani, Jessica Cherry, Farida Sohrabji

**Affiliations:** Women’s Health in Neuroscience Program, Department of Neuroscience and Experimental Therapeutics, Texas A&M University College of Medicine, Bryan, TX, United States of America; Massachusetts General Hospital/Harvard Medical School, United States of America

## Abstract

Ischemia-induced cerebral infarction is more severe in older animals as compared to younger animals, and is associated with reduced availability of insulin-like growth factor (IGF)-1. This study determined the effect of post-stroke IGF-1 treatment, and used microRNA profiling to identify mechanisms underlying IGF-1’s neuroprotective actions. Post-stroke ICV administration of IGF-1 to middle-aged female rats reduced infarct volume by 39% when measured 24h later. MicroRNA analyses of ischemic tissue collected at the early post-stroke phase (4h) indicated that 8 out of 168 disease-related miRNA were significantly downregulated by IGF-1. KEGG pathway analysis implicated these miRNA in PI3K-Akt signaling, cell adhesion/ECM receptor pathways and T-and B-cell signaling. Specific components of these pathways were subsequently analyzed in vehicle and IGF-1 treated middle-aged females. Phospho-Akt was reduced by ischemia at 4h, but elevated by IGF-1 treatment at 24h. IGF-1 induced Akt activation was preceded by a reduction of blood brain barrier permeability at 4h post-stroke and global suppression of cytokines including IL-6, IL-10 and TNF-α. A subset of these cytokines including IL-6 was also suppressed by IGF-1 at 24h post-stroke. These data are the first to show that the temporal and mechanistic components of post-stroke IGF-1 treatment in older animals, and that cellular components of the blood brain barrier may serve as critical targets of IGF-1 in the aging brain.

## Introduction

Ischemic stroke is a leading cause of death worldwide and a major cause of long-term disability in survivors. Stroke risk increases with age [1] and occurs more frequently in older women than men [2]. Poor recovery and long-term disability is greater in postmenopausal women as compared to men [3], [4] or young women [5]. Experimental studies confirm worse stroke outcomes in aged (16 months) female mice as compared to young (2–3 months) females [6], and this age-related severity is seen as early as middle age (10–12 months) in female rats [7].

Middle age represents a critical window of stroke susceptibility in females, specifically associated with declining ovarian function. Acyclic, middle-aged female rats have constitutive increases in blood brain barrier permeability [8] and reduced functional capacity of critical support cells such as astrocytes [9], likely contributing to their increased stroke severity. Paradoxically, estrogen replacement to middle-aged or aged female rats is not neuroprotective [7], [10]. However, declining gonadal hormone levels in aging females is also paralleled by reduction in other endocrine factors such as IGF-1 [11]. Our previous work has shown that circulating and parenchymal IGF-1 levels are lower in middle-aged females compared to younger females rats [12].

IGF-1 is widely considered neuroprotective in brain injury and stroke. Decreased IGF-1 levels are linked to increased risk [13] and worse functional outcome after ischemic stroke [14]. In young animals, exogenous IGF-1 reduces ischemic injury [15]–[17] and promotes neuronal survival and angiogenesis [18], [19]. IGF-1 is a mitogen and activates the PI3k/Akt pathway that mediates cell survival, however it’s role in ischemic middle-aged and aging animals is poorly studied, as are the mechanisms underlying IGF-1 actions in this group.

A novel strategy was used to identify potential neuroprotective mechanisms for IGF-1 in middle-aged rats, specifically, comparing microRNA (miRNA) expression profiles in the ischemic brain. MiRNA, a small non-coding RNA, are important regulators of mRNA transcript stability [20] and gene translation [21]. MiRNA have been used successfully as biomarkers for cancer, cardiovascular and neurologic diseases [22] including stroke [23] as well as therapeutic targets for these diseases in experimental models [24], [25]. In the present study, we report that a small cohort of miRNAs were significantly downregulated by IGF-1 in ischemic tissue from middle-aged females, and KEGG (Kyoto Encyclopedia of Genes and Genomes) analysis of putative miRNA gene targets implicated PI3K-Akt signaling pathways, as well as pathways related to endothelial and immune cell function. Evaluation specific components of these pathways showed that IGF-1’s neuroprotective actions in middle-aged females was preceded by improvement in the blood brain barrier and suppression of local inflammatory mediators, indicating a unique anti-inflammatory role for IGF-1 in the aging brain and the blood brain barrier as a novel target for IGF-1-mediated neuroprotection.

## Materials and Methods

Middle-aged (9–11 mo. weight range 325–350 g) female Sprague Dawley rats were purchased from Harlan Laboratories, IN. All animals were housed in an AALAC-approved facility, maintained on a constant photoperiod (12h light:dark cycles) and fed *ad libitum* with laboratory chow (Harlan Telkad 8604) and water. All animal procedures were performed in accordance with National Institutes of Health guidelines for the humane care of laboratory animals and approved by the Texas A&M University (TAMU) Institutional Animal Care and Use Committee. All surgical and terminal procedures were performed under anesthesia.

Animals that were included in these studies met our previously established criteria to be classified as reproductive senescent [8], [26], [27], namely 5+ previous pregnancies and current persistent diestrus. Persistent diestrus was determined by vaginal smears obtained daily for 3 weeks. A total of 61 females met the criteria for reproductive senescence and were included in the experiments. All animals were fitted with intracerebroventricular (icv) cannulas and were randomly assigned to receive either IGF-1 or saline. All animals were also subject to middle cerebral artery occlusion (MCAo) to mimic ischemic stroke. We observed 25% (16/61) mortality due to stroke, which is typical for this middle-aged group with the suture stroke model. Mortality was seen in both control and IGF-1 treated animals. Animals were assigned to either 4h or 24h survival. Experimental groups for analysis of infarct, blood brain barrier permeability and cytokine expression consisted of 6–8 animals each in the control and IGF-1 treated groups.

### Surgeries

Middle cerebral artery occlusion: Rats were anesthetized and maintained at 37°C on heating pads. Animals were subjected to middle cerebral artery occlusion (MCAo) via intraluminal suture. Briefly, after the neck region was shaved and the skin disinfected with ethanol, a ventral midline incision was made on the skin. Superficial fascia on the right side of the neck was dissected and the underlying muscles were blunt dissected to expose the right common carotid (CCA), external (ECA) and internal carotid arteries (ICA). The ECA was separated from the vagus nerve, and tied off distally with silk sutures after cauterizing the small branches. Microsurgical clamps were placed on CCA and ICA, a loose tie was placed on the ECA and the free stump of ECA was aligned with the ICA. A size 39 nylon 4.0 suture of 22 mm length with a silicon-coated round tip (Doccol Corp., CA) was inserted into ICA lumen through a small nick on the ECA between the two ties. The suture was advanced along the ICA until it reached the origin of the MCA (∼ 20 mm of suture) and secured in position with nylon ties. The intraluminal suture was maintained for 90 min. and, then withdrawn. Tissue perfusion rate was monitored using Laser-Doppler Flowmetry (Moor Instruments, UK) and the perfusion index was calculated for both ischemic and reperfusion time points. MCAo resulted in an 80% reduction of blood flow compared to the pre-occlusion rate and re-perfusion restored the perfusion index back to pre-occlusion levels.

### Intracerebroventricular infusion of IGF-1

Animals were placed in a stereotaxic instrument (David Kopf instruments, CA), a 28 gauge cannula was implanted into the right lateral ventricle using the co-ordinates – 1.0 mm posterior to bregma, –1.4 mm medial lateral, –3.5 mm from dural surface, as described by [28], and anchored in place with loctite 454 (Braintree Scientific, MA). Animals were allowed to recover for 1 week following cannula implantation and prior to stroke surgery. An Alzet osmotic minipumps (1003D, Alzet corp., CA, flow rate 1 μl/h) filled with human recombinant (hr) IGF-1 (R&D laboratories, 100 μg/ml) primed overnight placed into a subcutaneous pocket between the scapula and spine, after 45 min of ischemia. Previous studies have shown that IGF-1 is stable in Alzet minipumps for upto 7 days and the dose of IGF-1 was found to be effective [12], [29]. Control animals were infused with artificial CSF. All animals were terminated either 4h or 24h post-reperfusion.

### Infarct Analysis

Infarct volume analysis was determined using our previous procedures [12]. Briefly, the brain was removed from the cranium immediately after decapitation and sliced into 2 mm coronal sections using a brain matrix (Roboz, US). Brain slices were incubated in 2% 2,3,5-triphenyltetrazolium chloride (TTC, Sigma-Aldrich, MO) at 37°C. for 20 min and stained slices were photographed using an Olympus digital camera attached to a surgical microscope. Images were coded and infarct volume was measured using image analysis software, Image J (NIH, MD) by an experimenter who was blind to the codes. Total brain infarct was calculated from 3–4 slices (per animal) and is expressed as the ratio of infarct volume in the ischemic hemisphere to the total volume of the non-ischemic hemisphere.

### Molecular analyses:

RNA extraction: Brain tissue from the ischemic hemisphere of IGF-1 and vehicle-treated controls was used for RNA extraction. To each tube, containing 175 mg of brain tissue, 750 μl QIAzol master mix (800 μl QIAzol and 1.25 μl 0.8 μg/μl MS2 (carrier) RNA per sample) was added. Following a 5-minute incubation at room temperature (RT), 200 μl chloroform was added to each sample. After a 2-minute incubation at RT, samples were centrifuged at 12,000xg for 15 minutes at 4°C. The aqueous phase is then transferred to a fresh tube and mixed with ethanol (1.5 vol). The sample was then loaded on to a RNeasy Mini Spin Column and centrifuged for 30 seconds at RT, 13,000xg. After sequential washes in RWT and RPE buffers, the columns were transferred to a fresh tube and RNA eluted with 50 μl of DNase/RNase-free water. Sample purity was assessed by Nanodrop technology and a ratio of 1.8 was considered acceptable. Samples were stored at –20°C until use.

PCR Amplification: Template RNA (samples) were incubated with reverse transcriptase for 60 min at 42°C, followed by heat-inactivation of the enzyme (5 min at 95°C) and used immediately. cDNA was diluted 80-fold and then incubated with SYBR® Green master mix. Ten μl was dispensed to each well of the 384-well PCR plate. Plates were centrifuged at 192xg for 1min at 25°C before insertion into the thermalcycler (ABI Thermal Cycler 7900HT). An activation/denaturation step (95°C, 10 min) precedes 40 amplification cycles each at 95°C, 10 s, 60°C, 1 min, ramp-rate 1.6°C/s. Each microplate consists of 168 LNA-microRNA primer sets of plasma/serum relevant human miRNA and 7 reference microRNAs, for use with the ABI 7900HT instrument. MicroRNA on this panel are found in tissue and fluids and are culled from different diseases including various types of cancer, neurological disorders/neurodegenerative diseases, allergies/inflammation. Due to the multifactorial nature of stroke, this panel was considered the most appropriate. All primers are LNA modified which allows for uniform T*m*, and confers greater specificity, allowing for discrimination between miRNA sequences with single nucleotide differences. Normalization: Cycle Thresholds (CT) were determined for each miRNA in each sample. CT values for 5 reference miRNA in each sample were averaged and then subtracted from each of the 168 miRNA of interest (ΔCT). MicroRNA profiles were obtained from ΔCT values.

**Protein extraction:** Cortical and striatal tissue from the ischemic and non-ischemic hemisphere was dissected and stored frozen until use. Protein was extracted as described in [26], [30]. Briefly, tissues were homogenized in lysis buffer (50 mM TRis,pH 7.4, 150 mM NaCl, 1 mM EGTA, 1 mM Na-orthovanadate,pH 10, 5 μM ZnCl2, 100 mM NaF, 10 μg/ml leupeptin, 10% glycerol, 5 μM ZnCl2, 10 μg/ml aprotinin, 1 μg/ml leupeptin, 1 mM phenylmethylsulfonylfluoride in dimethylsulfoxide, and 1% Triton X-100) and centrifuged at 18000 rpm for 30 min. Supernatant was collected and stored at –20°C. until further analysis. Protein concentrations were determined using BCA protein assay kit (Pierce,IL).

### Western blot analysis

Western blotting was performed as described previously [12], [31]. Briefly, proteins (25 μg/lane) from the ischemic and non-ischemic hemisphere were size-fractionated on SDS-Page gels, and transferred to nylon membranes (Life Technologies, CA). Membranes were blocked and incubated with primary antibodies for either phosphorylated Akt or ERK (1∶200, SCBT, CA) followed by incubation with HRP-conjugated secondary antibodies (1∶2000, Transduction laboratories, KY). Blots were visualized using the Alpha Innotech imager in conjunction with the ECL detection method. Thereafter, the blots were stripped and reprobed with the pan-antibody for Akt and ERK. In each case, the intensity of ERK and Akt positive bands were quantified using FluorChem software, and phosphorylated protein signal was normalized to the appropriate pan antibody signal.

For detection of heavy (H) and light (L) chain IgG, proteins (25 μg/lane) from the ischemic and non-ischemic hemisphere were size-fractionated on SDS-Page gels, and transferred to nylon membranes (Life Technologies, CA) as before. Blots were incubated with HRP-conjugated anti-rat IgG antibody (1∶1000,Vector Laboratories, CA) and processed as above to visualize the immunoreactive signal. The IgG immunoreactive signal was quantified and intensity of the band of the ischemic hemisphere was normalized to the non-ischemic hemisphere.

### Multiplex bead cytokine assay

Tissue expression for a panel of cytokine/chemokine was measured in the ischemic and non-ischemic hemisphere of IGF-1 and vehicle treated animals, using a multiplexed magnetic bead immunoassay (Milipore Corp. MA) following manufacturer’s instructions. Briefly, the filter plate was blocked with assay buffer for 10 min and decanted. Standards and samples was added into appropriate wells, followed by addition of premixed beads and incubated for 2h at room temperature on a plate shaker. Wells were washed 2 times, 25 μl of detection antibody was added, incubated for 1h incubation at room temperature (RT) and followed by 30 min incubation with addition of 25 μl of streptavidin-phycoerythrin per well. After 2 washes, beads were resuspended in 150 μl of sheath fluid and a minimum of 50 beads per analyte was analysed in a Bio-Plex suspension array system (Bio-Rad Laboratories,CA). Cytokine/chemokine levels were normalized to total protein content. The following chemokines and cytokines were assessed: Granulocyte colony stimulating factor (G-CSF), eotaxin, GM-CSF, interleukin (IL)-1α, leptin, macrophage inflammatory protein (MIP)-1a, MIP2, IL-4, IL1-b, IL-2, IL-6, EGF, IL-13, IL-10, IL-12 phosphorylated 70 KDa (IL-12-p-70), Interferon (IFN)-γ, IL-5, IL-17A, IL-18, Chemokine C-motif Ligand (CCL2), Interferon gamma induced protein (IP)-10, Growth related oncogene/Keratinocyte derived chemokine (GRO/KC), VEGF, Fractalkine, Lipoploysacharide-induced CXC (LIX) chemokine, MIP-2, Tumor necrosis factor (TNF)- α, RANTES (regulated on activation, normal T cell expressed and secreted).

### Thiobarbituric Acid Reactive Substances (TBARS) Assay

Lipid peroxidation in the brain tissue was determined using the TBARS assay kit (Cayman chemical, MI) according to manufacturer’s instructions. Briefly, a mixture of 100 μl of sample, standard and 100 μl of SDS was first prepared. To this mixture, 4 ml of color reagent was added and boiled for an hour. The reaction was stopped on ice for 10 min and centrifuged for 10 min at 1600 g. The supernatant (150 μl) was loaded on a 96 well plate and absorbance was read at 540 nm in Tecan plate reader. TBARS concentration was calculated from a malondialdehyde (MDA) standard curve using Magellan software (CA) and normalized to the amount of total protein.

### Blood Brain Barrier Permeability Assay

Four hours following MCAo and reperfusion, animals were re-anesthetized and injected with Evan’s blue (2% at a volume of 4 mL/kg body weight) via the right jugular vein [8]. After 40 min, the animals were decapitated while under anesthesia. Brains were removed from the cranium, meninges were carefully removed and the brains were arranged in acrylic matrices (Braintree Scientific, MA) for sectioning. Brain slices between +1.7 to –1.4 mm from bregma [28] were collected. Cortical and striatal tissues were dissected separately from both the ischemic and non-ischemic hemispheres. Brain tissues were immediately weighed and dried in an incubator at 55°C. Dry tissues were weighed, homogenized in 50% trichloroacetic acid, centrifuged at 10,000 rpm for 10 min, and the supernatant was collected. Samples and standards containing known concentrations of Evan’s dye were loaded on a microplate and read at 620 nm (excitation); 680 nm (emission). The concentration of Evan’s blue was determined from a linear standard curve, using Magellan software (Tecan, Switzerland).

### Data analysis

MiRNA profile analysis: MicroRNA expression data (ΔCT) obtained from focus panels were uploaded into Genesifter® Analysis Edition (GSEA, Geospiza/Perkin Elmer Seattle, WA) software program. Differences in microRNA expression were identified by t-tests, with Benjamini and Hochberg correction for multiple comparisons at a cutoff α = 0.05. MicroRNA that were significantly regulated were graphically represented as a heat map.

Further *in silico* analysis was performed using DIANA-miRPath v2.0 (Vlachos et al., 2012), with the microT-CDS algorithm. Predicted and validated gene targets and the associated KEGG pathways were identified using a modified Fischer’s exact test with a FDR (Benjamini and Hochberg)-corrected p-value threshold of < 0.05.

Statistical analysis for other assays: Effect of IGF-1 on infarct volume and blood brain barrier permeability was analyzed by Student’s t-test. Two-way ANOVA were used to determine the effect of IGF-1 on inflammatory cytokines, TBARS, and expression of Akt and ERK. Each ANOVA was coded for treatment (IGF-1 or vehicle) as an independent variable and hemisphere (ischemic or non-ischemic) as a repeated measure, using the statistical package SPSS 21.0. Group differences were considered significant at p<0.05. Results are expressed as mean ± SEM.

### Results

IGF-1 reduces infarct volume in the acute phase of ischemia-reperfusion injury

**Figure 1:**
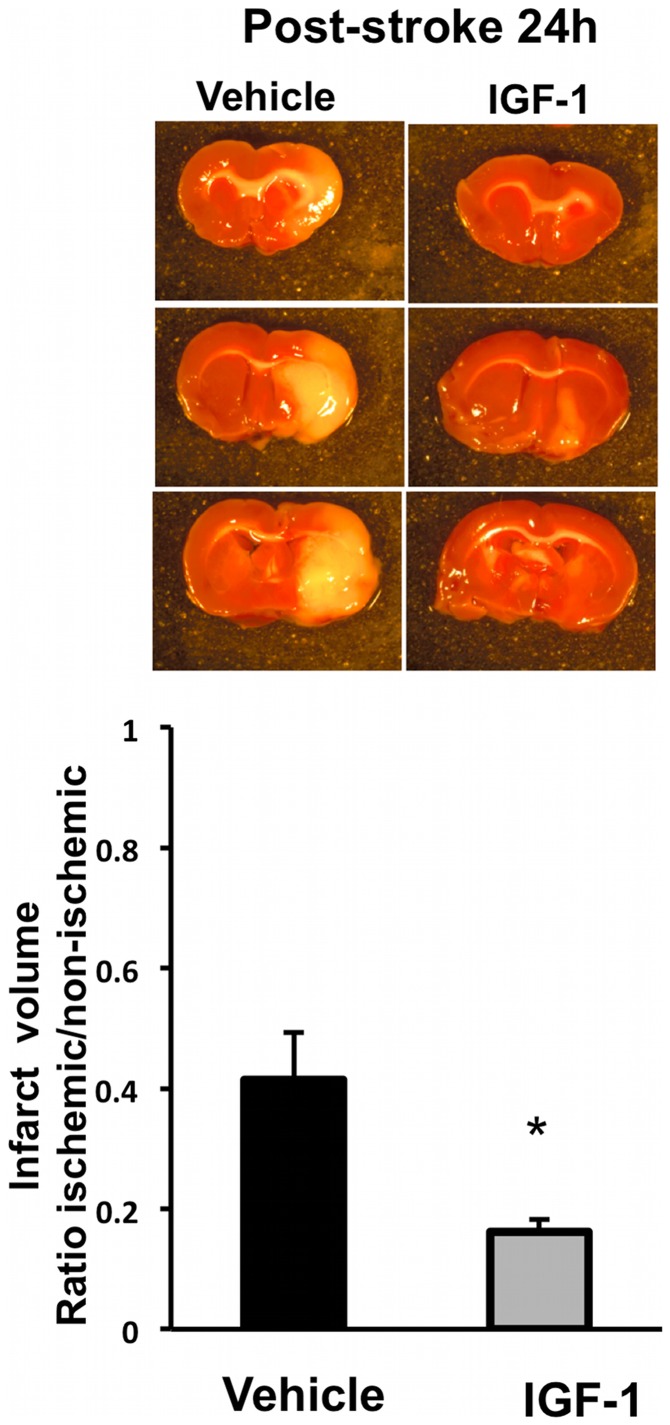
Effect of IGF-1 on infarct volume: MCAo for 90 min followed by reperfusion resulted in a cortical-striatal infarction as seen in TTC-stained coronal sections. Quantitative analysis of infarct volume, expressed as a ratio to the non-ischemic hemisphere, shows that post-stroke IGF-1 treatment resulted in a 39% decrease in infarct size compared to control after 24 h. Bars represent mean ± SEM. N =  6–8 in each group. *: p <0.05.

To determine the effect of IGF-1 treatment on brain infarct volume during the acute phase of stroke, female rats were subjected to MCAo occlusion and exposed to ICV-delivered IGF-1 or vehicle (artificial CSF, aCSF) treatment, immediately after reperfusion. At 24h post MCAo, IGF-1-treated middle-aged animals displayed a significant reduction (39%, p = 0.03) in infarct volume as compared to vehicle-treated females (Figure 1), indicating a significant neuroprotective effect for this peptide hormone.

### IGF-1 mediated neuroprotection: Mechanism of action

**Figure 2:**
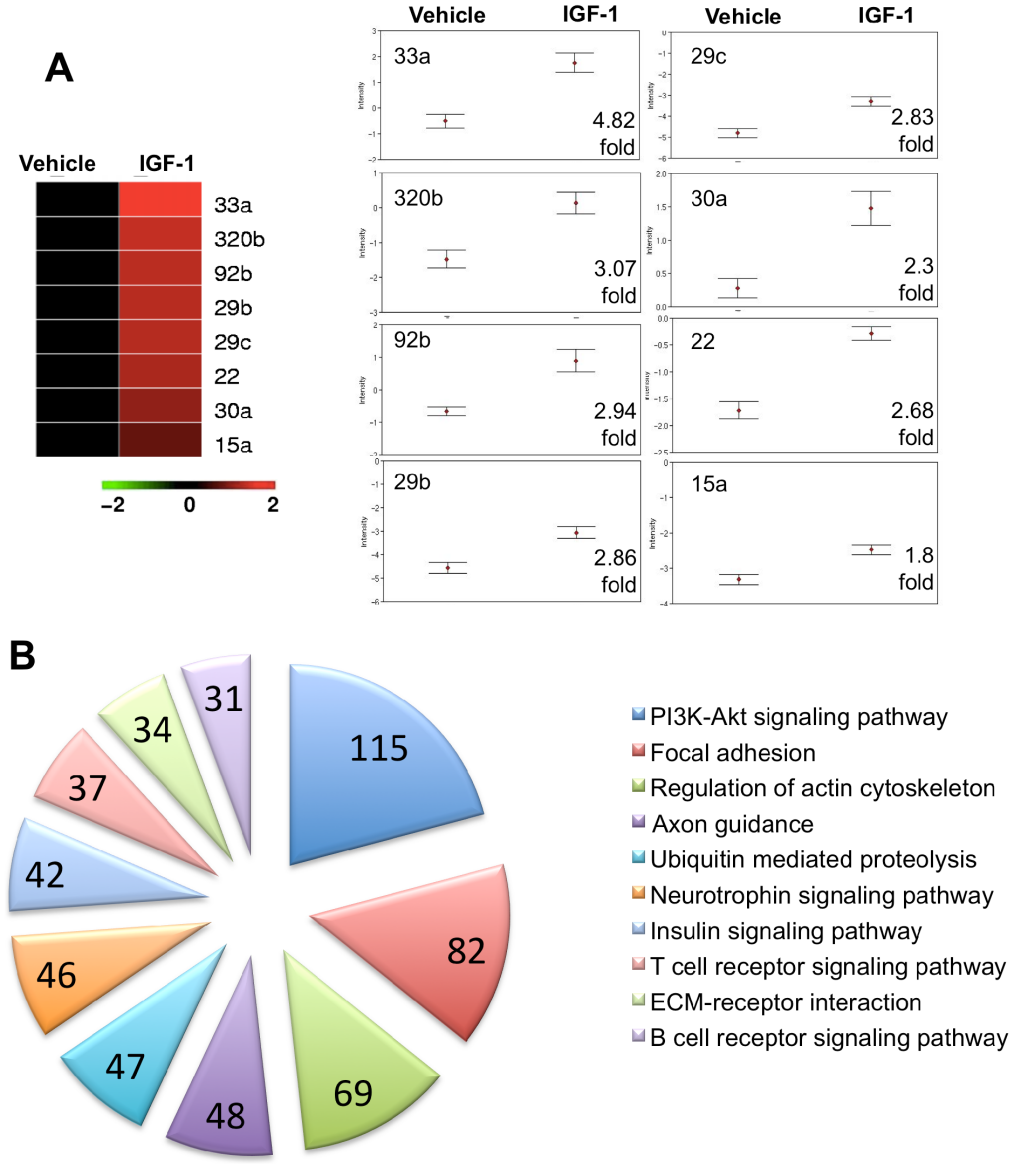
MiRNA regulation by IGF-1 in the ischemic brain at 4h post stroke. MiRNAs that were significantly regulated (adjusted for FDR) are graphically represented in a heat map format. Within the heat map, the vehicle-treated animals were used as ‘controls’ and are represented in black, while IGF-1 treated animals are depicted in relation to vehicle controls (columns). All miRNA in this cohort were significantly downregulated by IGF-1 (shown in red) in comparison vehicle controls (rows). (**B**) Group differences in expression patterns of IGF-1 regulated miRNA are shown graphically, with the ‘fold’ regulation indicated at the bottom right corner. Data (mean+SEM) are expressed are expressed as ΔCT, where an increased value indicates decreased miRNA expression. N  =  6/group p<0.05. (C) MiRNAs that were significantly regulated by IGF-1 treatment were subject to *in silico* analysis (DIANA/miRPATH, see methods). Predicted gene targets for each miRNA were identified. The top 10 KEGG pathways represented by these predicted targets are shown as a pie chart. Each slice represents the number of predicted target genes in the pathway, indicated within the slice. Each pathway is color coded and labeled adjacent to the chart. The top 10 pathways were selected based on the smallest “p” value and the largest number of target genes in the pathway.

MiRNA regulation by IGF-1: To identify neuroprotective mechanisms regulated by IGF-1, RNA from ischemic tissue from vehicle and IGF-1 treated animals was obtained at an early acute stroke phase (4h after MCAo), and interrogated using a focus panel of disease-related miRNA. QPCR analysis of a panel of 168 miRNA revealed that 8 miRNAs were significantly regulated by IGF-1 treatment after FDR (Figure 2A). These miRNA included mir33a, 320b, 92b, 29b, 29c, 22, 30a, 15a. Within this subset, IGF-1 decreased expression of all miRNA (Figure 2B), ranging from a 1.8–4.82 fold downregulation.

*In silico* analysis tools **(**DIANA-miRPath v2.0, and target database microT-CDS) was utilized to obtain predicted gene targets and associated Kyoto Encyclopedia of Genes and Genomes (KEGG) pathways of the IGF-1 regulated miRNAs. In total, 76 KEGG pathways were identified and from these, the 10 most relevant KEGG pathways are shown in Figure 2C. The top 10 pathways were selected based on the following criteria: smallest ‘p’ values for the union of common pathways targeted by all miRNAs, and the largest number of genes represented in those pathways, both indicators of robust pathways. This included the PI3-Akt signaling pathway, which is closely associated with cell survival and protection, and is also a principal component of the neurotrophin and insulin signaling pathways. A second large group of pathways identified by miRNA/KEGG analysis center around maintenance of cell structure, such as focal adhesion, actin cytoskeleton and ECM-receptor interactions, indicative of endothelial function. Finally, a third set of pathways regulate T and B cell signaling, cell types that are recruited to the brain following stroke. In subsequent studies, specific components or markers of these pathways were assessed in IGF-1 and vehicle-treated animals.

**Figure 3:**
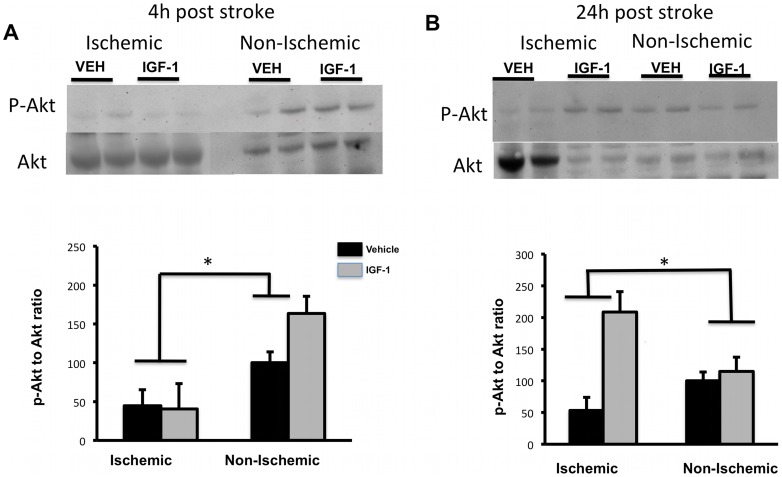
Phospho-Akt and Akt expression in post-ischemic brain. pAkt and pan-Akt levels were analyzed by Western blot (top panels). pAkt expression, normalized to pan-Akt, is shown in the histograms (lower panels). (A) p-Akt levels were reduced in both control and IGF-1-treated groups in the ischemic hemisphere at 4h, as compared to the non-ischemic hemisphere (main effect of ischemia; p<0.05). (B) At 24h, p-Akt expression was significantly increased in the ischemic hemisphere of the IGF-1-treated group as compared to the vehicle-treated group (interaction effect of ischemia and IGF-1). Bars represent mean ± SEM. n =  6 in each group. *: p <0.05.

*IGF-1 effect on survival kinases*: To test the Akt pathway, Western blot analysis was used to determine the effect of IGF-1 on the expression of phosphorylated-Akt and the related MAP kinase, ERK in brain lysates. At 4h post-stroke (Figure 3A), pAkt expression was reduced in the ischemic hemisphere (main effect of hemisphere, (F_(1,10)_ = 14.91;p = 0.003) in vehicle and IGF-1 treated animals. However, at 24h post stroke (Figure 3B) IGF-1 treatment significantly enhanced pAkt expression in the ischemic hemisphere (interaction effect, hemisphere x treatment; F_(1,10)_ = 22.49;p = 0.001).

**Figure 4:**
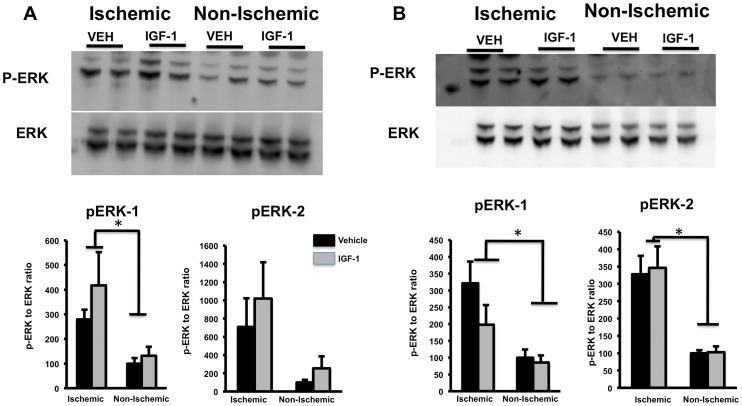
Phospho-ERK and pan ERK expression in post-ischemic brain. pERK and pan ERK were analyzed by Western blot (top panels). pERK expression, normalized to ERK, are shown in the histograms (lower panels). (A) p-ERK1 level increased in the ischemic hemisphere at 4h, as compared to the non-ischemic hemisphere (main effect of hemisphere, p<0.05). (B) At 24h, both p-ERK1 & 2 were significantly increased in the ischemic hemisphere (main effect of hemisphere; p<0.05). IGF-1 did not affect pERK expression at either time point. Bars represent mean ± SEM. n =  6 in each group. *: p <0.05.

Ischemia upregulated activated ERK-1 and ERK-2 at 4h (main effect of hemisphere, (F_(1.10)_ = 23.20;p = 0.001 & F_(1.10)_ = 10.74;p = 0.008 respectively; Figure 4A) while at 24 h, both ERK-1 and ERK-2 were elevated in the ischemic hemisphere (main effect of hemisphere, (F_(1.10)_ = 18.39;p = 0.002 & F_(1,10)_  = 41.62;p = 0.00007 respectively; Figure 4B). IGF-1, however, did not alter ERK phosphorylation in either hemisphere at the 4h and 24h time points (Figure 4A& B).

**Figure 5:**
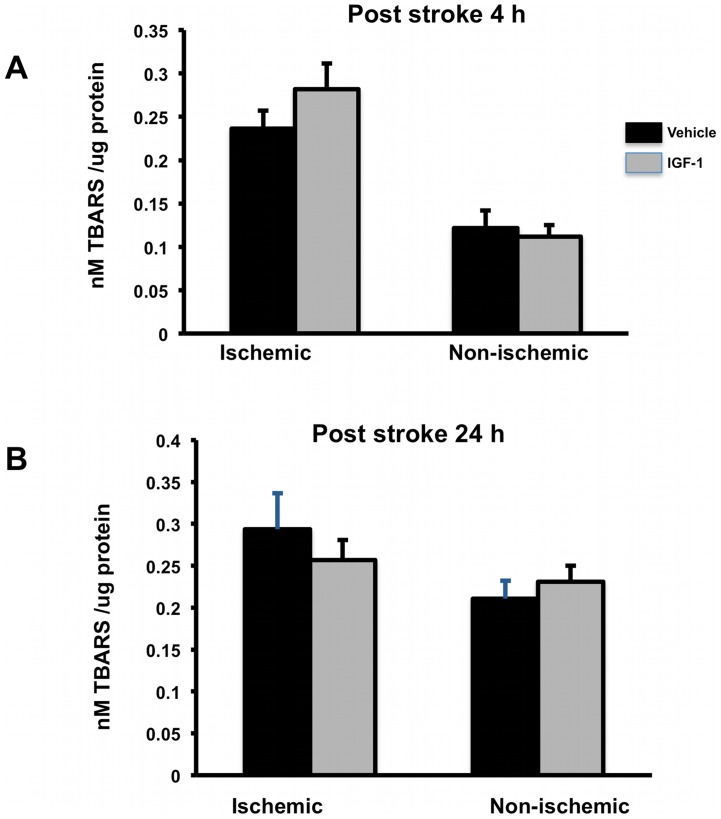
Expression of TBARS: (A) TBARS levels were increased with ischemia-reperfusion at 4h in both control and IGF-1 group compared to non-ischemic hemisphere. IGF-1 had no effect on TBARS levels. (B) At 24h post stroke, TBARS were similar in the ischemic and non-ischemic hemisphere and were not altered by IGF-1 treatment. Bars represent mean ± SEM. n =  6–8 in each group. *: p<0.05.

*Effect of IGF-1 on Oxidative stress:* Post-stroke cell death is characterized by rapid necrotic cell death with elevated levels of reactive oxygen species. We assessed whether IGF-1 altered the levels of thiobarbituric reactive species (TBARS), as a proxy measure of reactive oxygen species. As shown in Figure 5A, at 4h TBARS values were significantly higher in the ischemic hemisphere, indicative of ischemia-induced cell death seen in this hemisphere. However, IGF-1 did not attenuate TBAR levels in ischemic tissue. At 24h post-stroke (Figure 5B), there was no difference between the two hemispheres, indicating an abatement of reactive oxidative substances at this later time point. IGF-1 had no effect on TBARS at this time point as well.

**Figure 6:**
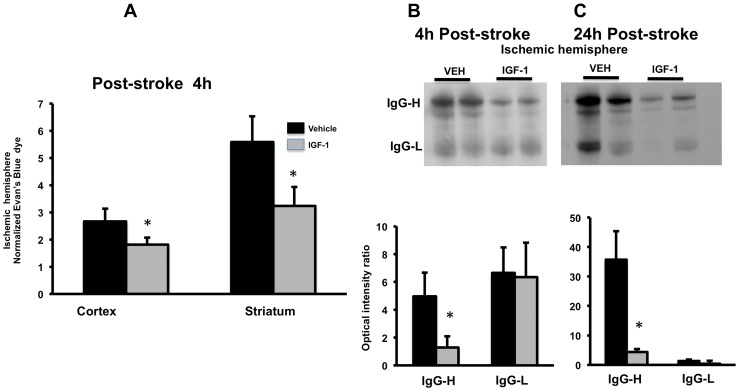
Effect of IGF-1 on ischemia-reperfusion induced blood brain barrier permeability. (A). Evans blue dye extravasation: IGF-1 treatment significantly reduced ischemia-induced increase in blood brain barrier permeability at 4h as indicated by decreased Evan’s blue dye in both cortex and striatum. Concentration of Evan’s blue is expressed as ratio of dye accumulation in the ischemic hemisphere to the non-ischemic hemisphere. Bars represent mean ± SEM. n =  6–8 in each group. *: p <0.05. (B) IgG expression: Western blot analysis of light and heavy chain IgG expression indicated that IGF-1 treatment reduced IgG-heavy chain expression at both 4h and 24h post stroke.

*IGF-1 effect on blood brain barrier permeability:* Blood brain barrier permeability was assessed by Evan’s blue extravasation and by IgG expression in brain tissue. For this study, the cortex and striatum were dissected and analyzed separately. A 2-way ANOVA indicated that the amount of dye extravasation was greater in the striatum as compared to the cortex (F_(1,14)_: 15.04; p = 0.002). However, IGF-1 treatment improved post-stroke barrier integrity in both regions (F_(1,14)_:5.97; p = 0.028), reducing dye accumulation by 62% in the cortex and 57% in the striatum (Figure 6A).

IgG expression was used as an additional surrogate marker for post-stroke changes in blood brain barrier function. Western blot analysis from ischemic brain lysates showed the presence of both heavy and light chain IgG at 4 & 24h time points (p = 0.001 and 0.003 respectively; Figure 6B & C). The expression of IgG-H in the ischemic hemisphere, normalized to the non-ischemic hemisphere, was significantly greater in the vehicle-treated group as compared to the IGF-1-treated group at both time points, supporting the conclusion that peptide treatment reduced blood brain barrier permeability.

**Figure 7:**
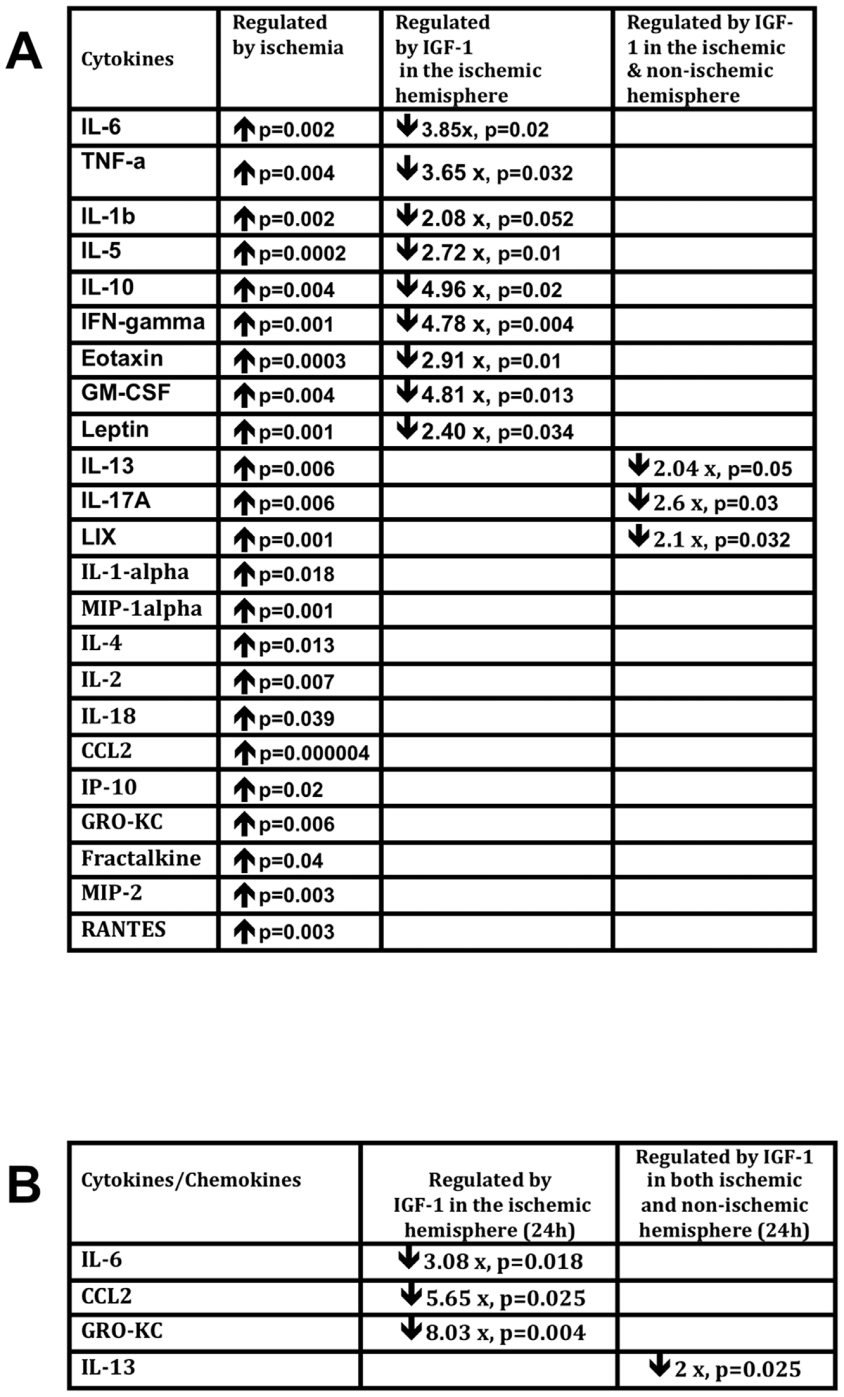
IGF-1 mediated regulation of inflammatory cytokines in post-ischemic brain. (A). Multiplex analysis of brain tissue lysates from the ischemic and non-ischemic hemisphere at 4h post stroke. The summary table shows each cytokine,and its regulation by ischemia or by IGF-1. Almost all cytokines were upregulated by ischemia. IGF-1 decreased the expression of approximately half of the cytokines on this panel (column 3; indicated by downward arrows and fold change), in the ischemic hemisphere and a few in both hemispheres (last column). (B) At 24 h, IL-6 and IL-13 continued to be suppressed in the IGF-1-treated group, as well as the chemokines GRO-KC and CCL2. n =  6-8 in each group; p<0.05 in each case.

*Effect on IGF-1 on cytokine and chemokine expression:* Ischemia triggers inflammation, which plays a major role in exacerbating stroke injury. A panel of cytokines and chemokines were examined in ischemic brain lysates obtained from animals terminated 4h and 24h post-stroke. At 4h post-stroke, virtually all cytokines were elevated in the ischemic hemisphere (Figure 7A; indicated by arrows). IGF-1 treatment caused a significant suppression of several pro- and anti-inflammatory cytokines (Figure 7A; indicated by downward arrows), ranging from a 2 to 5-fold suppression. In most cases, IGF-1 suppressed cytokines only in the ischemic hemisphere (interaction effect hemisphere x treatment). These included IL-6, TNF-a, IFN-g, IL1- b, GM-CSF, eotaxin, leptin, IL-5 and IL-10. In the case of IL-13, LIX and IL-17a, IGF-1 suppressed these cytokines in both hemispheres (main effect of treatment for each; Figure 7A, last column).

Cytokine expression was also examined at 24h post-stroke (Figure 7B) At this time point, IGF-1 treatment suppressed CCL2, IL-6 and GRO-KC in the ischemic hemisphere (interaction effect hemisphere x treatment) and IL-13 in both hemispheres (main effect of treatment). Both IL-6 and IL-3 were suppressed by IGF-1 at 4h as well, indicating a sustained action on specific inflammatory mediators.

## Discussion

These studies show that post-stroke IGF-1 treatment reduces infarct volume in middle-aged female rats. Using miRNA and putative target genes as a strategic tool, we show novel effects of IGF-1-mediated neuroprotection in an aging model. While IGF-1’s effects on Akt signaling are well known, this study demonstrates for the first time that IGF-1’s neuroprotective effects are preceded by an attenuation of stroke-induced blood brain barrier damage, concomitant with a rapid immunosuppressive effect in brain, and a sustained anti-inflammatory action.

Although miRNA have been used successfully as biomarkers [22], [23] as well as therapeutic targets [24], [25] for disease such as stroke, the present study employed a discovery-based model to identify potentially novel neuroprotective pathways for stroke. We identified 8 miRNA that were significantly downregulated by IGF-1 treatment, and used these miRNA to develop gene ontology patterns. Although gene ontologies suggest putative targets, and are incompletely validated, identification of the PI3K-Akt pathway through the miRNA/KEGG analysis underscores the promise of this approach. Akt signaling is a central component of neurotrophin and insulin signaling pathways and is known to promote cell survival, proliferation and differentiation [32]. The present data and published reports [16], [33] show that pAkt is attenuated following cerebral ischemia. Furthermore, IGF-1 has been shown to stimulate cell survival pathways [34], [35] in vitro and in disease models such as subarachnoid hemorrhage [36], neonatal hypoxia ischemia [37] hippocampal trimethytin toxicity [38] and Parkinson’s disease [29]. While IGF-1 mediated Akt activation has been shown in a neonatal model of hypoxia-ischemia [37], the present study is the first to show that IGF-1 elevates pAkt in an aging stroke model.

The miRNA-based pathways also indicated that structural elements of the cerebral microvasculature, such as focal adhesion, ECM-receptor interactions and actin cytoskeleton regulation, may be implicated in IGF-1 mediated neuroprotection. Several genes within these pathways include the integrins, and integrin-dependent downstream signaling proteins such Fyn, Rho-associated protein kinase (ROCK) and Rac1 [39]. Integrins constitute one the best-characterized ECM adhesion receptors of the brain microvasculature, including the blood brain barrier [40]. The blood brain barrier is often affected in stroke patients [41], [42] and in experimental stroke models in young [43], [44] and aging rodents [6], [45], [46]. Improvements in barrier function are shown to improve stroke outcome [47], [48]. Ischemia-reperfusion induces structural and molecular changes in the brain vasculature and adjacent cells, causing dysregulation of the barrier and promoting neural inflammation. Striatal infarction and stroke-mediated blood brain barrier dyregulation is much greater compared to the cortex, possibly due to the geometry of striatal arteries and inadequate collateral circulation to this region. IGF-1, however, significantly reduced blood brain barrier permeability in both the cortex and striatum, as shown by dye extravasation and IgG expression. The latter are the most frequently used assays for blood brain barrier permeability, however, newer analyses such as the use of size-graded dextrans would more precisely determine the extent of barrier permeability.

IGF-1 may protect the blood brain barrier of older animals by directly enhancing survival of endothelial cells, although such actions may be limited during the early phase of stroke. In the present study, IGF-1 did not promote Akt activation at the 4h time point. In fact, several studies have indicated that rapid cell death, which occurs in the early aftermath of ischemia, may hinder IGF-1 action. Reactive oxygen species, that accumulate in the early phase of ischemic injury, reduce pAkt [49] and impair IGF-1-mediated signaling. The elevated TBARS seen in the present study at 4h post-stroke is consistent with this hypothesis. Elevated levels of glutamate, typically seen following ischemic injury, have also been shown to reduce Akt activation *in vitro* with a loss of sensitivity to IGF-1 by alternate phosphorylation of the IGFR docking site [50]. Thus, IGF-1 may promote barrier integrity by alternate methods such as maintaining paracellular junctions. IGF-1 has been shown to increase transepithelial electrical resistance (a measure of barrier function) by regulating occludin and claudin-3 in submandibular gland cells [51], zona occludens (ZO)-1 in A431 human epidermoid carcinoma cells [52] and claudin-1 in osteoblast cells [53]. IGF-1 receptors have been identified on human microvascular endothelial cells [54] and aortic endothelial cells [55] although IGF-1’s effects on the molecular properties of the blood brain barrier are poorly understood. Specifically, analysis of brain microvessels and expression of junction proteins following IGF-1 treatment are urgently needed.

Dysregulation of the blood brain barrier coupled with the local inflammatory response triggers infiltration of circulating neutrophils, macrophages and T-cells [56], [57] which plays a cardinal role in the pathogenesis of cerebral ischemia particularly during the acute stage of stroke onset [58], [59]. Increased infarct volume in aged females, for example, is associated with greater T-cell infiltration as compared to young females and age-matched males [60]. Consistent with previous stroke studies [61], [62], we also observed an increase in several brain inflammatory cytokines including IL-6, TNF-α, IL-1β in the ischemic hemisphere of vehicle treated animals. IGF-1 treatment, however, exerted a profound suppressive action on the stroke-induced immune response as early as 4h, decreasing the expression of both pro and anti-inflammatory cytokines. At 24 hours post-stroke, IGF-1 treatment continued to inhibit selective pro-inflammatory markers, such as IL-6, the pleitrophic cytokine IL-13 and the chemoattractants CCL2 and GRO-KC. These findings argue that IGF-1 exhibits a biphasic action, an immediate, antigen non-specific or global immunosuppression followed by inhibition of specific pro-inflammatory cytokines at a later time point, culminating in reduced ischemic injury.

While the early global suppression of cytokine/chemokine expression by IGF-1 may be secondary to its effect on barrier integrity, the continued suppression of specific cytokines at the 24 hour time point suggests that IGF-may also exert a direct anti-inflammatory effect. KEGG pathway analysis indicated that IGF-1 regulated miRNA also impact T and B cell signaling, where key target genes include PDCD1, which negatively regulates the immune responses through the T-cell receptor (TCR) [63], and LYN, which encodes the tyrosine protein kinase LYN, an inhibitor of myeloid lineage proliferation [64], suggesting that IGF-1 regulation of miRNA may suppress immune cell proliferation and differentiation. However, little is known of IGF-1’s direct actions on cytokine expression in leukocytes, and in some cases, its actions appear to be contradictory. For example, in mast cells, IGF-1, acting via PI3-K, stimulates IL-6 and TNF-α while attenuating IL-1β [65] while in muscle cells, IGF-1 treatment reduces IL-6 and TNF-α via suppression of TLR4 [66]. An important future direction for this work is the direct assessment IGF-1 actions on T and B cell cohorts recruited to the ischemic brain.

The animal model used in these studies was intended to mimic an important demographic that is at a higher risk for stroke as well as stroke severity, namely middle-aged females, where ovarian hormones such as estrogen are reduced as well as other endocrine mediators such as IGF-1, thyroid hormone and Vitamin D. In middle-aged female rats, we have shown that estrogen treatment is not neuroprotective [7], [12], however estrogen treatment coupled with IGF-1 overcomes the neurotoxic effects of estrogen in this population [12]. The role of IGF-1 treatment alone on this estrogen-deficient population has not been explored previously. Collectively, these data suggest that post-stroke IGF-1 treatment improves stroke outcome by minimizing the impact of stroke on blood brain barrier disruption and by shaping the post-stroke neuroinflammatory profile.
